# Cyclic fatigue resistance of reduced-taper nickel-titanium (NiTi) instruments in doubled-curved (S-shaped) canals at body temperature

**DOI:** 10.34172/joddd.2020.024

**Published:** 2020-06-17

**Authors:** Gülşah Uslu, Mustafa Gundogar, Taha Özyurek, Gianluca Plotino

**Affiliations:** ^1^Department of Endodontics, Faculty of Dentistry, Çanakkale Onsekiz Mart University, Çanakkale, Turkey; ^2^Department of Endodontics, Faculty of Dentistry, Istanbul Medipol University, İstanbul, Turkey; ^3^Grande Plotino & Torsello Dental Clinic, Rome, Italy

**Keywords:** Double curvature, Fatigue resistance, Nickel-titanium, Rotary instruments

## Abstract

**Background.** This study was conducted to compare the cyclic fatigue resistance of VDW.ROTATE, TruNatomy Prime, HyFlex CM, and 2Shape nickel-titanium (NiTi) rotary instruments in double-curved canals in a simulated clinical environment.

**Methods.** Eighty NiTi files were used for the fatigue testing in stainless steel canals compatible with instrument sizes until fracture occurred (n=20): VDW.ROTATE (04./25#), TruNatomy Prime (04./26#), HyFlex CM (04./25#) and 2Shape TS04./25#( 1). For each instrument, the number of cycles to fracture (NCF) was calculated, and the fractured fragment length (FL) was measured. To verify that the files were fractured due to cyclic fatigue, the fractured surfaces of the files were evaluated under a scanning electron microscope. Data were statistically analyzed using the Kruskal–Wallis and Student’s t-tests at the %95 confidence level.

**Results.** The failure of the files due to cyclic fatigue was first seen in the apical curvature before the coronal curvature (P<0.05). The highest fatigue resistance was observed in VDW.ROTATE and HyFlex CM files in both curvatures (P<0.05). There were no significant differences in the fatigue resistance between the HyFlex CM and VDW.ROTATE files or between the 2Shape and the TruNatomy files (P>0.05). There was no difference in the fractured lengths of the files between the apical and coronal curvatures (P>0.05).

**Conclusion.** In artificial S-shaped root canals, VDW.ROTATE and HyFlex CM files exhibited higher fatigue resistance compared to 2Shape and TruNatomy files.

## Introduction

Despite technological advances in metallurgy and design, nickel-titanium (NiTi) files can unexpectedly fracture during clinical use, resulting in a poor prognosis of the tooth under treatment.^1^ The fracture of NiTi files can take place through torsional or cyclic fatigue.^2,3^ Fracture due to torsional fatigue occurs as a result of the file being stuck in the root canals while the handpiece continues to rotate. Cyclic fatigue occurs as a result of the freely rotating file within the canal being exposed to repeated cycles of compression and tensile forces at the most inclined part of the root canal.^4,5^ A previous study showed that the role of the clinician in cyclic fatigue failure is minimal.^6^ Instead, root canal curvature is the main factor involved in instrument failure by increasing flexural stresses on the file.^7^ S-shaped root canals are not detected on conventional radiographs, and in these canals, the stress on the file increases, making the treatment more difficult for clinicians.^8^

Minimally invasive endodontic treatment approaches aim to protect the tooth structure and root dentin as much as possible.^9^ It has been reported that conservative access cavity preparation with minimal tissue loss and root canal preparation made with small tapered instruments increases the fracture resistance of the teeth.^10-14^ New NiTi file systems incorporating instruments with small tapered designs have been introduced for conservative root canal preparation to protect the root structure and decrease the possibility of file separation in curved root canals.

One of these NiTi rotary systems, VDW.ROTATE (VDW, Munich, Germany), consists of three files for use in the preparation of narrow (#15, .04 taper; #20, .05 taper; and #25, .04 taper) or wide (#15, .04 taper; #20, .05 taper; and #25, .06 taper) root canals. The files have an S-shaped cross-section, off-center design, and constant taper. The files undergo a special heat treatment developed by the manufacturer.

The other system, TruNatomy (Dentsply Sirona, Ballaigues, Switzerland) NiTi rotary system, consists of five different files: #20, .08 taper (orifice modifier); #17, .02 taper (glide path); #20, .04 taper (small); #26, .04 taper (prime); and #36, .03 taper (medium). The files have a square cross-section and an off-centered design, with a variable taper. These files have four times more elasticity and fatigue resistance as compared with the file systems produced with the conventional heat treatment technique due to a new heat treatment procedure used as reported by the manufacturer.

HyFlex CM (Coltène/Whaledent, Altstätten, Switzerland) and 2Shape (Micro-Mega, Besancon, France) are NiTi files already on the market. These are composed of different heat-treated alloys, and both file systems have small tapered NiTi files (#25, .04 taper).^15^ 2Shape files have an asymmetrical cross-sectional design with a triple helix to reduce the risk of instrument fracture and enhance the cutting efficiency of the file. The files are produced with a symmetrical horizontal cross-section with three cutting edges, only #25, .04 taper files have four flutes and a square-shaped horizontal cross-section.^16^

A literature review showed no cyclic fatigue study about VDW.ROTATE or TruNatomy NiTi files. The evaluation of resistance to cyclic fatigue of VDW.ROTATE, TruNatomy Prime, HyFlex CM, and 2Shape files produced by different methods but with similar taper (.04) was performed at body temperature and in an aqueous environment in order to mimic the clinical conditions. The null hypothesis of the study was that there would be no difference in the fatigue resistance of the files tested at body temperature and in an aqueous environment.

## Methods

The sample size was calculated at n=20 per group based on the power analysis carried out by considering the data of a previous study. Thus, 80 files were selected for the present study, as follows: VDW.ROTATE (#25, .04 taper), TruNatomy Prime (#26, .04 taper), HyFlex CM (#25, .04 taper) and 2Shape TS1 (#25, .04 taper) NiTi files. All the files were inspected under a dental operation microscope (OMG 2350, Zumax Jiangsu, China) at ×20 magnification before the test to detect any deformation. No deformation was observed in the files examined; therefore, they were all used for cyclic fatigue testing.

Coronal and apical curvature parameters of the artificial double-curved stainless steel canals were as follows, respectively: 60° angle and 5-mm radius of curvature and 70° angle and 2-mm radius of curvature as described in a previous study.^17^ The centers of apical and coronal curvatures were 2 and 8 mm away from the tip of the root canal, respectively.

Then, the artificial root canals were immersed in distilled water in a glass container heated until the temperature of water reached the body temperature (37±1°C), using a hot plate. The temperature was continuously checked with an infrared thermometer during the test.^18^ Using a torque-controlled endodontic motor (Reciproc Gold; VDW) and considering the instructions of the manufacturers, NiTi rotary files were freely rotated in the root canals until fracture occurred. The VDW.ROTATE was rotated at 300 rpm and 2.3 Ncm; TruNatomy Prime was rotated at 500 rpm and 1.5 Ncm; HyFlex CM was rotated at 500 rpm and 2.4 Ncm; and 2Shape TS1 was rotated at 300 rpm and 2.5 Ncm.

The time until fracture was recorded in seconds by a stopwatch, and the number of cycles to failure (NCF) was calculated by multiplying the time by the rpm for each file. In order to verify that the files were positioned correctly in the artificial root canals and similar stresses affected the files, the fractured lengths (FL) of the files were measured using a micro-caliper.

Two fractured fragments from NiTi file groups were evaluated under a scanning electron microscope (JEOL, JSM-7001F, Tokyo, Japan) to characterize fracture modes. Images were captured of the fractured file surfaces under different magnifications (×100 to ×3,000).

### Statistical analysis

Statistical analyses were carried out using SPSS 21.0 (IBM, SPSS Inc., Chicago, IL, USA). Inter-group comparisons of NCF data of the NiTi files were made with the Kruskal-Wallis test. For inter-group comparisons of the FL data, one-way ANOVA and post hoc Tamhane test were performed. Student’s t-test was performed for intra-group comparisons of the NCF and FL data. Statistical significance was set at the 95% confidence level.

## Results

**Figure 1:**
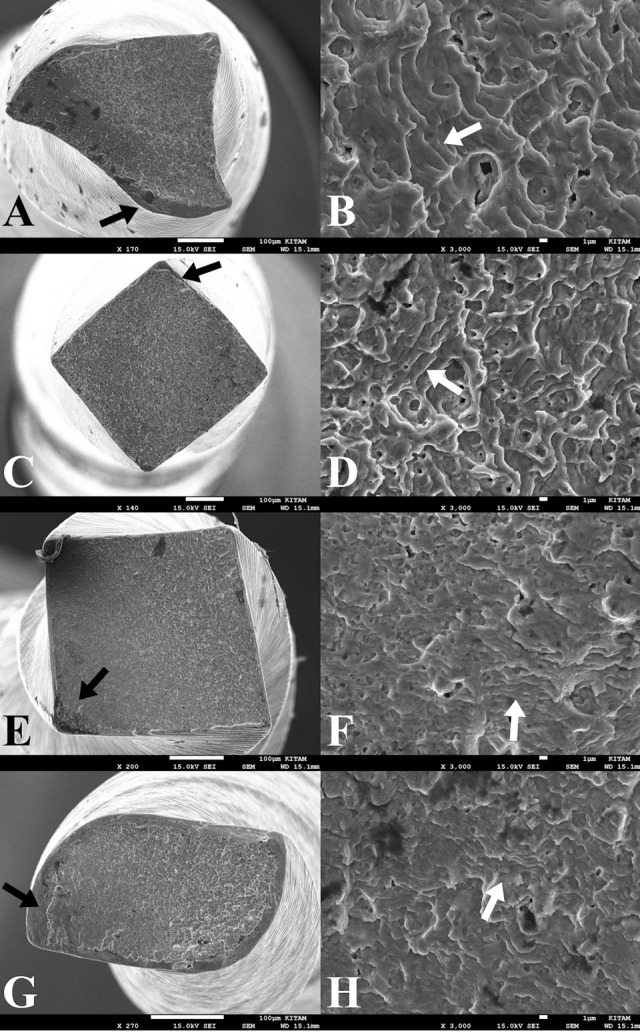
Scanning electron microscopic images of the fractured cross-section of (A) 2Shape, (C) TruNatomy, (E) HyFlex CM and (G) VDW.ROTATE and high-magnification view of (B) 2Shape, (D) TruNatomy, (F) HyFlex CM, and (H) VDW.ROTATE indicating fatigue striations peculiar to cyclic fatigue (arrows).

NCF and FL values of VDW.ROTATE, TruNatomy, HyFlex CM, and 2Shape NiTi files are listed in Table 1. NiTi file fractures were seen in the apical curvature before the coronal curvature (P<0.05). In both curvatures, VDW.ROTATE and HyFlex CM NiTi files exhibited significantly higher cyclic fatigue resistance than 2Shape and TruNatomy files (P<0.05). There were no significant differences in the fatigue resistance between VDW.ROTATE and HyFlex CM files and between 2Shape and TruNatomy files (P>0.05). Apical and coronal curvatures did not differ between the files in terms of the lengths of the fractured fragments (P<0.05). Scanning electron microscopic images did not show any signs of fracture due to cyclic fatigue of the files (Figure 1).

**Table:** Number of cycles to failure (NCF) and fragment length (FL, mm) of the fractured fragments of the different instruments tested during static cyclic fatigue testing in double curvature at body temperature.

		**Apical Curvature**			**Coronal Curvature**			
**Group**	**n**	**NCF**	**FL**	**NCF**	**FL**	***P*** **-value**
**VDW.ROTATE**	20	475.65 ± 56.15 ^bx^	2.36 ± 0.52 ^a^	580.32 ± 71.25 ^by^	8.34 ± 1.04 ^a^	< .05
**TruNatomy**	20	329.75 ± 41.05 ^ax^	2.39 ± 0.57 ^a^	375.43 ± 58.13 ^ay^	8.12 ± 1.17 ^a^	< .05
**HyFlex CM**	20	471.50 ± 55.05 ^bx^	2.73 ± 0.55 ^a^	575.67 ± 89.49 ^by^	8.44 ± 1.32 ^a^	< .05
**2Shape**	20	309.37 ± 40.72 ^ax^	2.43 ± 0.52 ^a^	365.65 ± 61.62 ^ay^	8.05 ± 1.21 ^a^	< .05
***P*** **-value**		< .05	> .05	< .05	> .05	

*Different superscript letters indicate significant difference at 5% level (^a,b,c^ for columns; ^x,y^ for rows).

## Discussion

The vast majority of teeth have curved root canals when examined in different planes, especially in the buccolingual aspect.^19^ The root canal anatomy of teeth cannot be determined from a proximal view on standard radiographs.^20^ Instead, the root canal curvatures can be detected only by cone-beam computed tomography (CBCT).^21^ The treatment of teeth with dilacerated (S-shaped) root canals can be challenging because file breakage and loss of working length are frequently reported as complications in such teeth.^22^ This study aimed to investigate the cyclic fatigue resistance of NiTi files in double-curved root canals.

According to a previous study, it is important to assess a file’s trajectory during cyclic fatigue tests.^23^ Thus, in the present study, each artificial root canal was manufactured compatible with instruments’ size and taper. Although the vast majority of studies on S-shaped root canals have been conducted at room temperature, some studies showed that different ambient temperatures influenced the fracture resistance of heat-treated NiTi instruments.^18,24-27^ Cyclic fatigue testing of the files was carried out at body temperature in this study to simulate clinical conditions similar to that in a study by Huang et al.^18^ However, the frequent replacement of the irrigants used during the preparation and the contact of the NiTi files with the irrigants in the root canals might cause the canal temperature to vary depending on the temperature of the solution used. This is important when the ambient temperature to which NiTi files are exposed is considered as a factor in cyclic fatigue studies.

Previous cyclic fatigue studies of NiTi files employed static or dynamic models.^28-30^ In static models, the file is operated at a constant length, without moving it in the axial direction. In dynamic models, it is operated using a forward-backward movement at the maximum curvature point. Thus, the cyclic fatigue resistance of files is higher in dynamic models.^30^ Keleş et al^31^ evaluated the cyclic fatigue of Reciproc Blue (VDW), Reciproc (VDW), WaveOne Gold (Dentsply Sirona), and WaveOne (Dentsply Sirona) files in static and dynamic models at room and body temperatures. They reported that axial movement increased the cyclic fatigue of the files when a dynamic model was used, whereas similar results were obtained when the file systems were tested using a static model. Although the dynamic model aims to simulate how files are operated in root canals, the model cannot replicate actual clinical conditions. In the present study, to exclude various effective factors in cyclic fatigue, a static model was used to test the NiTi files similar to that in other studies.^32,33^

The results of the present study showed that in both the apical and coronal curvatures, the fatigue resistance of VDW.ROTATE and HyFlex CM files was significantly higher than that of TruNatomy and 2Shape files. Thus, the null hypothesis was refuted. VDW.ROTATE and TruNatomy are new file systems. As there are no studies on their cyclic fatigue resistance in the literature, the results of the present study cannot be directly compared with those of previous research. As is well known, the heat treatment used in the manufacture of NiTi files can significantly affect the fatigue resistance of the files.^29^ HyFlex CM files are produced with the well-known controlled memory (CM) heat treatment technology, which significantly increases the fatigue resistance of the files.^28,34^ In this study, the fatigue resistance of HyFlex CM files produced from CM-Wire was found to be higher than that of TruNatomy and 2Shape files.

The size of the cross-sectional area of a file might also influence the file’s mechanical properties, with a larger horizontal cross-sectional area, resulting in enhanced flexural and torsional hardness.^35^ Several studies reported that an S-shaped cross-section provided improved cyclic fatigue resistance at the maximum curvature area because of its lower metal mass.^7,34^ The S-shaped horizontal cross-section of VDW.ROTATE files might have contributed to its enhanced resistance to cyclic fatigue in this study.

In artificial root canals, the diameter of the instrument at the maximum curvature area affects the cyclic fatigue resistance of the tested file.^7^ Thus, flexibility and fatigue resistance decrease as the instrument size and diameter increase.^36^ Apical diameters (0.25 mm vs. 0.26 mm) and tapers (variable vs. constant taper) of the files used in this study were not similar. The larger tip diameter of the TruNatomy file (#26 vs. #25), as compared with that of the other files, might account for its reduced cyclic fatigue resistance.

In the literature, there is a limited number of studies on the cyclic fatigue resistance of NiTi files in S-shaped canals at body temperature. Elnaghy and Elsaka^37^ analyzed the cyclic fatigue resistance of One Curve (Micro-Mega), 2Shape, Vortex Blue (Dentsply Sirona), ProFile Vortex (Dentsply Sirona), and RaCe (FKG Dentaire, La Chaux-de-Fonds, Switzerland) files in single- and double-curved root canals and reported that 2Shape and One Curve files had similar cyclic fatigue resistances, but Vortex blue files had higher fatigue resistance compared to these two files.

According to the results of this study, all the files fractured mainly in the apical curvature (P<0.05), which might be justified by the severity of the curvatures, with the apical curvature having a radius of 2 mm and the coronal curvature having a radius of 5 mm. The results of previous cyclic fatigue studies on S-shaped root canals corroborate this idea.^8,17,22,38^

No difference was found between the files in terms of the mean FL of the files in the apical or coronal curvature in the present study. However, the mean FL in the apical curvature was shorter than that of the coronal curvature because of the curvature location, consistent with the results of several previous studies.^17,37,38^

The analysis of the fractured file surfaces by scanning electron microscopy demonstrated the ductile nature of the fracture, characterized by microscopic dimples. Higher magnifications showed that the fatigue lines were characterized by the crack progression in initial stages of fracture (Figure ?).

Since the production methods, heat treatments, and geometrical designs of the files can be effective in the results obtained from this study, the interpretation of the effect of a single factor is the limitation of this study as stated in other in vitro cyclic fatigue resistance tests of NiTi files.^39^

## Conclusion

In this in vitro study, the fatigue resistance of file systems with similar tapers (4%) was investigated under standardized clinical conditions (S-shaped canals and body temperature). Within the limitations of the study, the fatigue resistance of the VDW.ROTATE and HyFlex CM files was higher than that of the TruNatomy and 2Shape files. However, caution is needed in applying these findings to the clinical practice, as there is no single factor determining the file fracture under real conditions.

## Acknowledgement

None declared.

## Authors’ Contributions

GU, MG, TÖ, and GP designed the study. The methods section was actualized by GU, TÖ and MG. Data were statistically analyzed by TÖ and GP. The manuscript was written by GU and MG. TÖ and GP revised the manuscript.

## Conflict of Interests

The authors confirm that they have no conflict of interests.

## Funding

Not applicable.

## Ethics Approval

This article does not contain any studies with human participants or animals.
